# Anticipated First Contact and Time to Seek Healthcare for Possible Breast Cancer Symptoms: A Cross‐Sectional Study in Rural Ghana

**DOI:** 10.1111/hex.70395

**Published:** 2025-08-28

**Authors:** Adwoa Bemah Boamah Mensah, Kofi Boamah Mensah, Anita Eseenam Agbeko, Emmanuel Kwaku Nakua, Joshua Okyere, Gloria Anane, Joseph Sakyi Baah, Madalyn Nones, Keith J. Horvath, Beth Virnig, Joe‐Nat Clegg‐Lamptey, Shalini Kulasingam

**Affiliations:** ^1^ School of Nursing and Midwifery, College of Health Sciences Kwame Nkrumah University of Science and Technology Kumasi Ghana; ^2^ Department of Pharmacy Practice, College of Health Sciences, Faculty of Pharmacy and Pharmaceutical Sciences Kwame Nkrumah University of Science and Technology Kumasi Ghana; ^3^ Department of Surgery Komfo Anokye Teaching Hospital Kumasi Ghana; ^4^ Department of Epidemiology and Biostatistics, School of Public Health Kwame Nkrumah University of Science and Technology Kumasi Ghana; ^5^ School of Human and Health Sciences University of Huddersfield, Queensgate Huddersfield UK; ^6^ Department of Health Policy, Management and Economics, School of Public Health Kwame Nkrumah University of Science and Technology Kumasi Ghana; ^7^ Presbyterian Nursing and Midwifery Training College Asante‐Akyim Agogo Ghana; ^8^ Division of Epidemiology and Community Health University of Minnesota Minneapolis Minnesota USA; ^9^ Department of Psychology San Diego State University San Diego California USA; ^10^ College of Public Health and Health Professions University of Florida Gainesville Florida USA; ^11^ Department of Surgery University of Ghana Medical School Accra Ghana; ^12^ Celia Scott Weatherhead School of Public Health and Tropical Medicine Tulane University New Orleans Louisiana USA

**Keywords:** breast cancer, Ghana, health‐seeking behaviour, public health, rural, women

## Abstract

**Background:**

Breast cancer is a serious public health concern in Ghana. This study investigated the anticipated healthcare‐seeking behaviour and preferences of women for possible breast cancer symptoms. Specifically, the study examined women's first point of contact as well as the timeliness of seeking healthcare for possible breast cancer symptoms.

**Methods:**

This cross‐sectional survey data involved 810 women in 14 rural communities in the Ashanti region, Ghana. The outcome variables were preferred first contact and timing for healthcare seeking. Descriptive analysis and two sets of binary logistic regression models were used.

**Results:**

The results indicate that 58.9% of the participants would first contact a medical doctor, whereas 41.1% would contact alternative sources (herbalist/traditional healer, fetish priest/faith healer and pastor/Man of God). Almost all respondents (95.6%) anticipated seeking care early. Women who anticipated contacting alternative sources (e.g., herbalists, pastors or family members) over medical doctors had 2.58 times higher odds of anticipating delays in their healthcare seeking (AOR = 2.58, 95% CI: 1.28–5.21). Compared to women in agricultural/farming, those who worked in civil/government/private sectors had 7.87 times higher odds of anticipated delays in seeking healthcare (AOR = 7.87, 95% CI: 2.11–29.40). Women with tertiary education had 92% lower odds of anticipated delays (AOR = 0.08, 95% CI: 0.01–0.84). Additionally, women who based their healthcare decisions on advice from others had 66% lower odds of delaying healthcare seeking (AOR = 0.34, 95% CI: 0.14–0.85).

**Conclusion:**

Almost all women would seek healthcare early for possible breast cancer symptoms; however, a significant proportion anticipated contacting alternative/non‐medical sources. There is a need to raise awareness among women about the importance of consulting a medical doctor for breast cancer symptoms to ensure timely and appropriate medical assessment and treatment. Addressing hurdles such as limited access to medical facilities and financial constraints can improve health‐seeking preferences for breast cancer symptoms.

**Public Contribution:**

At the start of the project, a community‐based project advisory board (CPAB) was set up. The CPAB included local government representatives from the Ghana Health Service (*n* = 1) and District Assembly (*n* = 4), traditional leaders (*n* = 3), public sector health services (health service providers and managers) (*n* = 4), community women representatives (*n* = 4), breast cancer survivor (*n* = 1) and breast health advocates (*n* = 2). In this study, the CPAB members assisted in gaining community access, recruiting local women as study recruitment links in each site and providing guidance on field work safety. The study results have been discussed with CPAB members and field workers in a series of project meetings.

**Trial Registration:**

Not applicable.

**Abbreviations**
AOR: adjusted odds ratio
BCa: breast cancer
BSE: breast self‐examination
CHPS: Community‐Based Health Planning and Services
COR: crude odds ratio
LMICs: low‐and‐middle‐income countries
NCDs: non‐communicable diseases
PMA: Performance Monitoring for Action

## 1 Background

Globally, breast cancer remains the most common cancer, affecting approximately 7.8 million women at the end of 2020 [1]. It is estimated that, globally, breast cancer cases will increase by 33.8% between 2020 and 2040 [2], making breast cancer a serious public health concern. Breast cancer is also the most common malignancy in Ghana, with an estimated incidence of 76 cases per 100,000 women [3]. Furthermore, the breast cancer 3‐year survival rate in Ghana stands at 52% [4]. This suggests that nearly 48% of Ghanaian women diagnosed with breast cancer are likely to die within 3 years' post‐diagnosis. The low survival rate has partly been linked to delays in seeking healthcare and late diagnosis [5, 6].

To reduce breast cancer‐related mortalities and improve survival rates, it is imperative to understand the healthcare‐seeking behaviour of women. This is because the stage of diagnosis significantly predicts cancer outcomes, and late‐stage cancers (Stages III and IV) are the least responsive to curative treatment [7, 8]. Moodley et al. [8] assert that reducing the time to diagnosis hinges significantly on women's comprehension of breast cancer symptoms and risk factors, accurately assessing potential cancer symptoms, promptly seeking care from primary healthcare providers, receiving appropriate evaluation at the primary healthcare level and accessing referral and treatment centres promptly. Yet, there is a paucity of scientific evidence on women's anticipated preferences and the timeliness of seeking healthcare for possible breast cancer symptoms in Ghana.

Available evidence on breast cancer‐related healthcare‐seeking behaviours has focused on patients already diagnosed with the disease [9, 10, 11], which raises the potential of recall bias. Moreover, it makes the results of such studies non‐generalisable among women who are unaware of their breast cancer status. Consequently, there is a need for studies that focus on women with no history of breast cancer and their healthcare‐seeking behaviours to inform tailored and policy‐driven interventions. However, examining anticipated healthcare‐seeking behaviours captures real‐time preferences and intentions, which are less susceptible to memory distortions. To the best of our knowledge, there is currently no published study in Ghana that has attempted to fill this knowledge gap. Hence, our study aims to investigate the anticipated healthcare‐seeking behaviour for possible breast cancer symptoms. Specifically, the study examines women's first point of contact as well as the timeliness of seeking healthcare for possible breast cancer symptoms.

## 2 Materials and Methods

### 2.1 Study Design and Setting

We conducted a cross‐sectional study involving women aged 25 years and above in one rural setting: Onwe of the Ejisu Municipal Assembly, located in the Ashanti Region of Ghana. We used an initial cross‐sectional design to obtain information on women's anticipated healthcare‐seeking preferences and behaviour for symptoms suggestive of breast cancer. The study consisted of women from 14 sites: Donaso, Abenase, Edwenase, Achinakrom, Achinase, Sarpeh, Deduako, Donyina, Asienimpong, Timeabu, Kwaso, Sunsuaso, Onwe and Odaho. These communities are served by four health facilities, including two hospitals and two Community‐Based Health Planning and Services (CHPS). Onwe has a total of 100,384 households and 37,745 women aged 25–79 years at risk of developing breast cancer [12]. However, none of the health facilities within the locality offer breast cancer screening or diagnostic services. Consequently, women in this area commonly travel at least an hour to the city for breast health services [13].

### 2.2 Participants, Sampling and Sample Size

We targeted resident women of Onwe, a rural sub‐metro of the Ejisu Municipal Assembly. Eligibility criteria included women aged 25 years or older, without a history of breast cancer and residing in the designated rural communities under investigation. Individuals aged < 25 years were excluded from this due to the low incidence of breast cancer among younger women [2]. Participants were required to be fluent in either Twi or English, the primary languages of the region and provide informed consent by writing/thumb‐printing before participation. At each site, households were selected using a multistage cluster sampling methodology. The existing household sampling frame from the Onwe sub‐metro area, established for the Performance Monitoring for Action (PMA) initiative, served as the basis for sampling. PMA is a comprehensive nationwide household survey conducted through healthcare facilities that monitors various health indicators, including family planning uptake [14]. Within the PMA platform, households within each enumeration area are listed, mapped and systematically sampled for inclusion in survey rounds, employing random selection methods. One woman per identified household was then randomly selected and invited to participate in the study. We initially calculated an estimated sample size of 940 and had 914 women enrolled, thus, a 97.2% response rate. Overall, 88.6% (*n* = 810) of women had no missing observations with respect to the anticipated healthcare provider preference and timing for healthcare seeking. These women were the focus of our study. This sample is representative of the Onwe sub‐metro in the Ejisu Municipal Assembly.

### 2.3 Recruitment and Consent

The study team gained permission from the study sites with an introductory letter and copies of an ethical approval letter, following which a pre‐data collection interaction on the study with the Municipal assembly and each community leader was conducted. Recruitment outlets were the communities within the Onwe sub‐metro. Following random sampling of households from the PMA frame, trained research assistants (RAs) worked with local community recruitment links to locate the sampled households and facilitate contact with eligible participants. Women who met the eligibility criteria were then informed about the study and invited to participate. If a woman expressed interest, she was met at her house where the RA provided detailed information about the study and conducted a thorough eligibility assessment. Once eligibility was confirmed, and if the woman was willing to participate after receiving all necessary study details, informed consent was obtained by writing, and the questionnaire was administered. Recruitment of participants took place from 14 March 2023 to 31 May 2023.

### 2.4 Study Variables

#### 2.4.1 Outcome Variables

The main outcome variable was anticipated time for healthcare seeking. The participants were asked, ‘How long do you usually wait to report at the clinic when symptoms appear?’ The responses for this question were the following: the same day or immediately I notice symptom or sign, within 1 week of symptom or sign, within 1 month but less than 3 months and more than 3 months of a symptom or sign. Seeking healthcare immediately or within 1 week of noticing symptoms was classified as ‘Early healthcare‐seeking behaviour’, whereas seeking healthcare within 1 month or more was categorised as ‘Late healthcare‐seeking behaviour’. This categorisation was informed by an earlier study [8].

#### 2.4.2 Explanatory Variables

In this study, we defined ‘anticipated first contact’ as the type of healthcare provider or person a participant may first contact with a breast problem for help. This was derived from the question, ‘Who will be your first contact person for help when you notice a problem with your breast?’ This question had the following responses: medical doctor, drug store/community pharmacy staff, herbalist/traditional healer, fetish priest/faith healer, pastor/Man of God and friend/family member. All responses other than contacting a medical doctor were categorised as ‘alternative contacts’. Informed by previous studies [8, 9, 10, 11], we selected other explanatory variables: age (i.e., 25–29, 30–34, 35–39, 40–44, 45–49 and 50+ years), educational level (i.e., no formal education, primary, secondary and tertiary), occupation (i.e., agriculture/farming, self‐employed, housewife, civil/government/private and other), marital status (i.e., never married, married/cohabiting, separated/divorced and widowed), income (< GHS500, GHS500–999 and ≥ GHS1000), distance to healthcare facility as a problem and autonomy in decision‐making. Autonomy in decision‐making was derived from the question, ‘I can make my own decisions regarding health care’. Participants were also asked what primarily influenced their decisions to seek healthcare when needed. This included whether they considered their financial capacity, whether they had active health insurance or if they sought advice from others (e.g., family or community members) before making health‐related decisions.

### 2.5 Ethical Approval

This study follows the Declaration of Helsinki and the Belmont Declaration. Ethical approval was obtained from the Ghana Health Service Ethics Review Committee (GHS‐ERC: 016/01/23).

### 2.6 Data Collection Tool and Technique

We adopted and adapted the validated African Awareness of Cancer (AWACAN) tool [15], which is available in English. The AWACAN tool was modified and expanded with context‐specific items from a previous qualitative study [16]. These revised items were reviewed by three public health researchers in Ghana and pretested among 30 rural women from a nearby community not included in the main study to evaluate cultural relevance, clarity and translation accuracy. Data were collected by trained graduate RAs using handheld electronic tablets. The questionnaire was programmed in Research Electronic Data Capture (REDCap) to enable real‐time data collection and uploading, as well as to assist with quality control and efficient data processing [17]. Women were interviewed face‐to‐face at their homes in either the local language (Twi) or English, depending on their preference. Each questionnaire took approximately 35 min to complete.

### 2.7 Statistical Analysis

Following data collection, the information was extracted from the REDCap system and imported into STATA Version 18 (StataCorp, College Station, TX, USA) for both data management and subsequent analysis. The initial step involved data cleaning procedures, which entailed removing all instances of missing data (*n* = 104) (11.4%) for all variables included in the analysis. Descriptive analysis was then conducted to examine the frequency distribution of various baseline characteristics within the sample. Additionally, the proportion of women's anticipated healthcare‐seeking preferences and behaviours was estimated. Subsequently, both bivariable and multivariable logistic regression models were applied to explore the factors associated with women's anticipated healthcare‐seeking preferences and behaviour. In the bivariable analysis, results were presented as crude odds ratios (CORs) along with their corresponding 95% confidence intervals (CIs). For the multivariable analysis, adjusted odds ratios (AORs) and 95% CI were reported, accounting for potential confounding factors. A backward stepwise approach was used to select variables for the multivariable logistic regression. Only variables that had a *p*‐value < 0.05 were included in the final model.

## 3 Results

### 3.1 Participants' Profile

The majority were married or in cohabitation relationships (62.0%), were self‐employed (53.0%) and earned < GHS500 (75.7%), while 25.7% were women aged 50 years and above. Most participants had attained secondary education (58.4%). Regarding healthcare decisions, 64.7% relied on health insurance, and a substantial 77.9% made decisions based on their financial status. Additionally, 35.7% based their healthcare decisions on advice from others. While 47.7% considered distance to healthcare facilities a problem, 59.7% lacked autonomy (required permission from others) in healthcare decision‐making (see Table 1).

**Table 1:** Background characteristics of participants.

Variables	Sample (*n*)	Proportion (%)
*Overall*	810	100.0
*Age*		
25–29 years	179	22.1
30–34 years	140	17.3
35–39 years	105	12.9
40–44 years	94	11.6
45–49 years	84	10.4
50+ years	208	25.7
*Marital status*		
Never married	131	16.2
Married/cohabitation	502	62.0
Separated/divorced	96	11.8
Widowed	81	10.0
*Occupation*		
Agricultural/farming	224	27.6
Self‐employed	429	53.0
Housewife	30	3.7
Civil/government/private	80	9.9
Other	47	5.8
*Education*		
No formal education	145	17.9
Primary	135	16.7
Secondary	473	58.4
Tertiary	57	7.0
*Healthcare decision based on health insurance*		
Yes	524	64.7
No	286	35.3
*Healthcare decision based on financial status*		
Yes	631	77.9
No	179	22.1
*Healthcare decision based on advice from others*		
Yes	289	35.7
No	521	64.3
*Distance to healthcare facility as a problem*		
Yes	386	47.7
No	424	52.3
*Autonomy in decision‐making*		
Yes	326	40.3
No	484	59.7
*Income*		
< GHS500	613	75.7
GHS500–999	132	16.3
≥ GHS1000	65	8.0

### 3.2 Anticipated First Contact and Anticipated Time to Seek Healthcare

**Figure 1:**
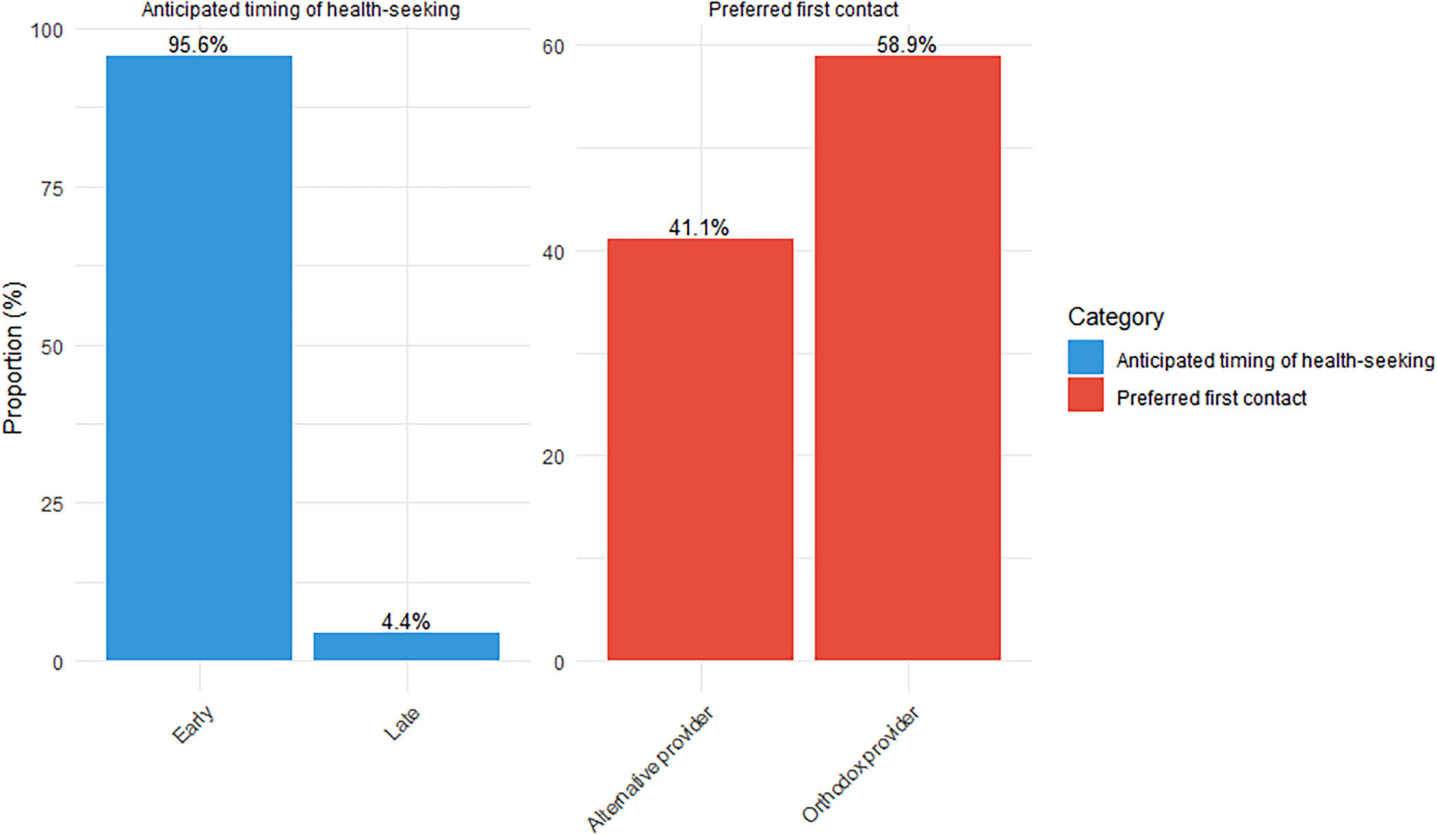
Anticipated first contact and timing for healthcare seeking.

Figure 1 presents the person or type of healthcare provider participants will first contact for possible breast cancer symptoms as well as the timing for healthcare seeking. The results indicate that 58.9% of the participants would first contact a medical doctor, whereas 41.1% anticipated contacting alternative sources. Also, most of the respondents (95.6%) would seek healthcare early (i.e., immediately or within 1 week of noticing symptoms).

### 3.3 Descriptive Distribution of the Anticipated Time to Seek Healthcare

Table 2 shows that individuals who anticipated contacting alternative or non‐medical care were more likely to seek healthcare late (7.2%) compared to those who preferred medical doctors (2.5%). Additionally, those whose healthcare decisions were not influenced by others' advice were more likely to seek healthcare late (5.9%) compared to those who were influenced (1.7%).

**Table 2:** Descriptive distribution of the anticipated time to seek healthcare.

Variables	Anticipated time to seek healthcare		*p* values
	Early *n* (%)	Late *n* (%)	
*Anticipated first contact*			0.001
Alternative/non‐medical	309 (92.8)	24 (7.2)	
Medical doctor	465 (97.5)	12 (2.5)	
*Age*			0.106
25–29 years	166 (92.7%)	13 (7.3%)	
30–34 years	136 (97.1%)	4 (2.9%)	
35–39 years	102 (97.1%)	3 (2.9%)	
40–44 years	87 (92.6%)	7 (7.4%)	
45–49 years	83 (98.8%)	1 (1.2%)	
50+ years	200 (96.2%)	8 (3.8%)	
*Marital status*			0.140
Never married	125 (95.4%)	6 (4.6%)	
Married/cohabitation	481 (95.8%)	21 (4.2%)	
Separated/divorced	88 (91.7%)	8 (8.3%)	
Widowed	80 (98.8%)	1 (1.2%)	
*Occupation*			0.433
Agriculture/farming	218 (97.3%)	6 (2.7%)	
Self‐employed	409 (95.3%)	20 (4.7%)	
Housewife	28 (93.3%)	2 (6.7%)	
Civil/government/private	74 (92.5%)	6 (7.5%)	
Other	45 (95.7%)	2 (4.3%)	
*Education*			0.234
No formal education	138 (95.2%)	7 (4.8%)	
Primary	125 (92.6%)	10 (7.4%)	
Secondary	455 (96.2%)	18 (3.8%)	
Tertiary	56 (98.2%)	1 (1.8%)	
*Healthcare decision based on health insurance*			0.646
Yes	502 (95.8%)	22 (4.2%)	
No	272 (95.1%)	14 (4.9%)	
*Healthcare decision based on financial status*			0.695
Yes	602 (95.4%)	29 (4.6%)	
No	172 (96.1%)	7 (3.9%)	
*Healthcare decision based on advice from others*			0.005
Yes	284 (98.3%)	5 (1.7%)	
No	490 (94.1%)	31 (5.9%)	
*Distance to healthcare facility as a problem*			0.078
Yes	374 (96.9%)	12 (3.1%)	
No	400 (94.3%)	24 (5.7%)	
*Autonomy in decision‐making*			0.225
Yes	315 (96.6%)	11 (3.4%)	
No	459 (94.8%)	25 (5.2%)	
*Income*			0.995
< GHS500	586 (95.6%)	27 (4.4%)	
GHS500–999	126 (95.5%)	6 (4.5%)	
≥ GHS1000	62 (95.4%)	3 (4.6%)	

### 3.4 Factors Associated With Longer Anticipated Time to Seek Care for Possible Breast Cancer Symptoms

In the unadjusted model, women who anticipated contacting alternative sources (e.g., herbalists, pastors or family members) over medical doctors were three times as likely to anticipate a longer time to seek healthcare (AOR = 3.01, 95% CI: 1.48–6.11). This association remained statistically significant after controlling for the other explanatory variables (AOR = 2.58, 95% CI: 1.28–5.21). Compared to women in agricultural/farming, those who worked in civil/government/private sectors had 7.87 times higher odds of anticipated delays in seeking healthcare (AOR = 7.87, 95% CI: 2.11–29.40). Women with tertiary education had 92% lower odds of anticipated delays (AOR = 0.08, 95% CI: 0.01–0.84). Additionally, lower odds of anticipated delays in healthcare seeking were found among women who based their healthcare decisions on advice (AOR = 0.34, 95% CI: 0.14–0.85) (see Table 3).

**Table 3:** Factors associated with longer anticipated time to seek care.

Variables	Crude model (COR, 95% CI)	Adjusted model (AOR, 95% CI)
*Anticipated first contact*		
Medical doctor	Ref.	Ref.
Alternative contacts	3.01 [1.48–6.11]**	2.58 [1.28–5.21]**
*Age*		
25–29 years	Ref.	—
30–34 years	0.37 [0.12–1.18]	—
35–39 years	0.38 [0.10–1.35]	—
40–44 years	1.03 [0.39–2.67]	—
45–49 years	0.15 [0.02–1.19]	—
50+ years	0.51 [0.21–1.26]	—
*Marital status*		
Never married	1.09 [0.43–2.78]	—
Married/cohabitation	Ref.	—
Separated/divorced	2.08 [0.89–4.85]	—
Widowed	0.29 [0.04–2.16]	—
*Occupation*		
Agricultural/farming	Ref.	Ref.
Self‐employed	1.78 [0.70–4.49]	2.12 [0.77–5.86]
Housewife	2.59 [0.49–13.48]	3.13 [0.59–16.54]
Civil/government/private	2.94 [0.92–9.41]	7.87 [2.11–29.40]**
Other	1.61 [0.32–8.26]	2.59 [0.51–13.27]
*Education*		
No formal education	Ref.	Ref.
Primary	1.58 [0.58–4.27]	1.31 [0.44–3.90]
Secondary	0.78 [0.32–1.90]	0.41 [0.15–1.12]
Tertiary	0.35 [0.04–2.93]	0.08 [0.01–0.84]*
*Healthcare decision based on health insurance*		
Yes	Ref.	—
No	1.17 [0.59–2.33]	—
*Healthcare decision based on financial status*		
Yes	1.18 [0.51–2.75]	—
No	Ref.	—
*Healthcare decision based on advice from others*		
No	Ref.	Ref.
Yes	0.28 [0.11–0.72]**	0.34 [0.14–0.85]*
*Distance to healthcare facility as a problem*		
Yes	0.53 [0.26–1.08]	—
No	Ref.	—
*Autonomy in decision‐making*		
Yes	0.64 [0.31–1.32]	—
No	Ref.	—
*Income*		
< GHS500	Ref.	—
GHS500–999	1.03 [0.42–2.56]	—
≥ GHS1000	1.05 [0.31–3.56]	—

*Note:* (—) variables removed after backward stepwise modelling.

Abbreviation: Ref., reference category.

* *p* < 0.05;

** *p* < 0.01.

## 4 Discussion

Early healthcare‐seeking behaviour is imperative to effective management and control of breast cancer. The present study examined women's first point of contact as well as their anticipated timeliness in seeking healthcare for possible breast cancer symptoms. We found that 95.6% women anticipated seeking healthcare early (i.e., within 1 week of noticing symptoms). The high prevalence of early healthcare‐seeking behaviour is consistent with Moodley et al.'s study [8], which reported that 82.7% of women in rural Uganda anticipate seeking healthcare within the first week of noticing possible breast cancer symptoms. The observed proportion of women who anticipate seeking healthcare early is higher compared to a reported 35.9% in South Africa [18].

The study revealed that 41.1% anticipated first contacting alternative contacts (i.e., drug store staff, herbalists/traditional healers, pastor, family and friends). This is inconsistent with Moodley et al.'s study [8] found that approximately 88% of women anticipated discussing possible breast cancer symptoms with someone close to them. In Ghana, public health initiatives and awareness campaigns, which are often led by hospitals, NGOs and community health educators, have increasingly focused on encouraging women to seek prompt biomedical care for breast symptoms. These campaigns highlight the benefits of early detection and hospital treatment, and explicitly caution against relying solely on herbalists, prayer camps or drug stores as first‐line responses. This targeted messaging has been reinforced through radio, television, church events and community outreach programs, aiming to reshape perceptions, health‐seeking intentions and norms [16].

Although only 4.4% of the participants anticipated delays in seeking healthcare, individuals who anticipated contacting alternative sources as their first contact were 2.58 times more likely to anticipate delays than those whose anticipated first contact was medical doctors. This means that despite the higher odds of anticipated delay among those who indicated non‐medical contacts, the majority within this group (91.5%) still anticipated seeking care early. This nuance suggests that the association between anticipated first contact and delay is not deterministic and warrants careful interpretation. It is plausible that women who anticipate delay for structural or personal reasons may seek support from informal sources while navigating their health challenges [19]. Conversely, women embedded in robust social networks may turn first to trusted individuals, not out of resistance to medical care, but as part of a culturally normative consultation process. Therefore, the observed relationship may reflect broader patterns of social embeddedness, perceived barriers or marginalisation, rather than a direct causal pathway.

We found that higher educational attainment reduced the odds of anticipated delays in seeking healthcare. This is in contrast with a related multicenter study conducted in the United Kingdom that found no statistically significant association between level of education and anticipated delays in seeking healthcare for breast cancer [20]. A plausible explanation could be that higher education likely equips women with enhanced health literacy, problem‐solving skills and greater access to resources [13]; thus, enabling them to better navigate health systems and overcome barriers that may contribute to delays in seeking or receiving care. Furthermore, educated women may have higher self‐efficacy and a better understanding of the importance of timely care, as well as greater confidence in their ability to advocate for their health needs.

Our study also revealed a significant association between employment type and anticipated delays in seeking healthcare. Specifically, women working in civil, government or private sectors were 7.8 times more likely to anticipate delays compared to women engaged in agricultural or farming activities. Time constraints may be a possible explanation for this. Women in formal employment often face rigid work schedules and limited flexibility [21, 22], making it challenging to take time off for medical appointments without jeopardising their job security or productivity. Perceived urgency could be another explanation for the observed disparity. This is in the sense that women in physically demanding farming roles might prioritise healthcare due to the immediate impact of health issues on their ability to work, whereas those in desk‐based or less physically demanding jobs might delay seeking care, perceiving their health concerns as less urgent.

Our study also showed that women who based their healthcare decisions on advice from others had 66% lower odds of delaying healthcare seeking. This finding was unexpected, especially when our study had established that preference for non‐medical persons as first contact was associated with longer anticipated time to seek healthcare. The observed association corroborates Poteat et al.'s study [23] that found a 32% lower odds of delays in breast cancer healthcare seeking among individuals who had sufficient social support. Women who base their healthcare decisions on advice from others may feel more emotionally reassured and confident in their decisions to seek care, reducing hesitation or fear associated with healthcare visits.

### 4.1 Implications for Policy and Practice

We observed that 41.1% indicated alternative/non‐medical sources as their first contact. This highlights a need for targeted interventions to address the factors influencing women's first contact choice for possible breast cancer symptoms. Public health initiatives should focus on improving awareness about the importance of early medical consultation for breast cancer symptoms, emphasising the role of timely diagnosis in improving treatment outcomes. It also underscores a need to engage alternative contacts (i.e., traditional healers, drug store staff and community leaders) as allies in promoting timely referrals to formal healthcare services. Our findings suggest that integrating health literacy into education curricula and adult community programs can amplify the protective effect of education. Given the significant association between employment type and anticipated time to seek healthcare, formal workplace policies must prioritise flexibility (e.g., paid medical leave, telehealth access and on‐site clinics) to address systemic constraints in formal sectors. These efforts must be accompanied by structural interventions to address access and resource‐related barriers. For instance, improving geographic access to breast health services in rural areas, subsidising transportation costs, expanding community‐based diagnostic services and integrating early detection into community health platforms (e.g., CHPS) can help translate intentions into actual early care.

### 4.2 Strengths and Limitations

The multistage cluster sampling from which the sample was drawn guarantees the representativeness and generalisability of our findings. Nonetheless, the cross‐sectional design of the survey inherently limits the ability to establish causal relationships between variables, as data are collected at a single time point. Additionally, the reliance on self‐reported data, particularly regarding anticipated time to seek healthcare and preferred first contact may have introduced social desirability bias. To reduce the desirability bias, we conducted the surveys in private without the presence of other family or community members. We continuously assured the participants that their responses were going to be confidential and that they would not be linked to their identities. Nevertheless, there may still have been some unconscious desirability bias; therefore, this should be noted. Because we measure anticipated (hypothetical) behaviour, our associations may not directly translate to actual outcomes; thus, policy suggestions are based on intentions, not proven behaviour change. Additionally, if unmeasured factors like social support influence both anticipated first contact and timing, the observed relationship may be partly due to confounding. We explicitly mention these points to caution readers that the results show associations under specific conditions. Nonetheless, it provides evidence of what is expected to inform interventions. The study did not capture other important factors such as the level of social support and social embeddedness. As such, we urge caution in interpreting the observed associations.

### 4.3 Conclusion

In conclusion, almost all participants anticipated seeking healthcare early for possible breast cancer symptoms; however, almost half anticipated contacting alternative/non‐medical sources rather than medical doctors. As such, efforts should be made to raise awareness among women about the importance of consulting medical doctors for breast cancer symptoms to ensure timely and appropriate medical assessment and treatment. This could involve targeted health education campaigns, community outreach programs and collaborations with traditional and alternative healthcare providers to promote a coordinated approach to breast cancer care. Additionally, addressing barriers such as limited access to medical facilities, financial constraints and cultural beliefs surrounding healthcare seeking is essential to facilitate women's access to timely and appropriate medical care for breast cancer symptoms.

## Author Contributions

**Adwoa Bemah Boamah Mensah:** conceptualization, methodology, investigation, data curation, formal analysis, funds acquisition, project lead and administration, writing – original draft, writing – review and editing. **Kofi Boamah Mensah:** conceptualization, methodology, investigation, data curation, formal analysis, funds acquisition, writing – review and editing. **Anita Eseenam Agbeko:** methodology, investigation, data curation, formal analysis, funds acquisition, writing – review and editing. **Emmanuel Kwaku Nakua:** conceptualization, methodology, investigation, data curation, formal analysis, funds acquisition, writing – review and editing. **Joshua Okyere:** methodology, investigation, data curation, software, formal analysis, writing – original draft, writing – review and editing. **Gloria Anane:** methodogy, data curation, writing – review and editing. **Joseph Sakyi Baah:** methodology, investigation, data curation, writing – review and editing. **Madalyn Nones:** methodology, data curation, writing – review and editing. **Keith J. Horvath:** conceptualization, project supervision (co‐international mentor), methodology, writing – review and editing. **Beth Virnig:** conceptualization, project supervision (co‐international mentor), methodology, writing – review and editing. **Joe‐Nat Clegg‐Lamptey:** conceptualization, methodology, investigation, data curation, formal analysis, project supervision (local mentor), writing – review and editing. **Shalini Kulasingam:** conceptualization, methodology, investigation, data curation, formal analysis, project supervision (international mentor), writing – review and editing.

## Ethics Statement

This study follows the Declaration of Helsinki and the Belmont Declaration. Ethical approval was obtained from the Ghana Health Service Ethics Review Committee (GHS‐ERC: 016/01/23). Participants received an information sheet outlining the study's purpose, duration, potential benefits, risks and discomforts. They were assured that their personal identities would remain confidential and that study data would be shared solely among the research team, and only with a third party if legally required. Data were encrypted and stored with password protection to prevent unauthorised access.

## Consent

Oral and written informed consent was obtained from all the participants.

## Conflicts of Interest

The authors declare no conflicts of interest.

## Data Availability

The data for the study are not publicly available due to ethical issues. The data are, however, available upon reasonable request from the lead author.
